# Computational biogeographic distribution of the fall armyworm (*Spodoptera frugiperda* J.E. Smith) moth in eastern Africa

**DOI:** 10.1016/j.heliyon.2023.e16144

**Published:** 2023-05-15

**Authors:** Elfatih M. Abdel-Rahman, Emily Kimathi, Bester Tawona Mudereri, Henri E.Z. Tonnang, Raphael Mongare, Saliou Niassy, Sevgan Subramanian

**Affiliations:** International Centre of Insect Physiology and Ecology (*icipe*), P.O. Box 30772-00100, Nairobi, Kenya

**Keywords:** Pest ecology, MaxEnt, Landscape, Climate change, Invasive species, Pest management, Citizen science

## Abstract

The fall armyworm (FAW), *Spodoptera frugiperda* J.E. Smith, has caused massive maize losses since its attack on the African continent in 2016, particularly in east Africa. In this study, we predicted the spatial distribution (established habitat) of FAW in five east African countries viz., Kenya, Tanzania, Rwanda, Uganda, and Ethiopia. We used FAW occurrence observations for three years i.e., 2018, 2019, and 2020, the maximum entropy (MaxEnt) model, and bioclimatic, land surface temperature (LST), solar radiation, wind speed, elevation, and landscape structure data (i.e., land use and land cover and maize harvested area) as explanatory variables. The explanatory variables were used as inputs into a variable selection experiment to select the least correlated ones that were then used to predict FAW establishment, i.e., suitability areas (very low suitability – very high suitability). The shared socio-economic pathways, SSP2-4.5 and SSP5-8.5 for the years 2030 and 2050 were used to predict the effect of future climate scenarios on FAW establishment. The results demonstrated that FAW establishment areas in eastern Africa were based on the model strength and true performance (area under the curve: AUC = 0.87), but not randomly. Moreover, ∼27% of eastern Africa is currently at risk of FAW establishment. Predicted FAW risk areas are expected to increase to ∼29% (using each of the SSP2-4.5 and SSP5-8.5 scenarios) in the year 2030, and to ∼38% (using SSP2-4.5) and ∼35% (using SSP5-8.5) in the year 2050 climate scenarios. The LULC, particularly croplands and maize harvested area, together with temperature and precipitation bioclimatic variables provided the highest permutation importance in determining the occurrence and establishment of the pest in eastern Africa. Specifically, the study revealed that FAW was sensitive to isothermality (Bio3) rather than being sensitive to a single temperature value in the year. FAW preference ranges of temperature, precipitation, elevation, and maize harvested area were observed, implying the establishment of a once exotic pest in critical maize production regions in eastern Africa. It is recommended that future studies should thus embed the present study's modeling results into a dynamic platform that provides near-real-time predictions of FAW spatial occurrence and risk at the farm scale.

## 1 Introduction

Globally, maize (*Zea Mays* L.) supports the food and nutrition requirements of about 900 million people [1, 2]. In sub-Saharan Africa (SSA), maize is one of the most planted crops among other cereals, principally grown by small-scale farmers for subsistence and income generation [3]. However, maize productivity in SSA has significantly declined in the past decade [4]. This is mainly attributable to a combination of yield constraints, including low adoption and uptake of modern maize production technologies, insect pests, diseases, weeds, climate change, moisture stress, low soil fertility, and archaic cultural practices, among others [[5], [6], [7]]. Consequently, depending on the region, these constraints account for approximately 21%–53% of maize yield losses annually in SSA [8] with arthropod pests being among the most devastating maize yield constraints causing combined yield losses of approximately 31% [9].

The fall armyworm (FAW); *Spodoptera frugiperda* (J.E. Smith), which is indigenous to the north and south America, has vigorously invaded most SSA countries since 2016 [10] exceeding the effects of other already existing endemic pests and causing maize yield losses of 33%–100% across the entire African continent [11]. Economically, annual losses in crops like maize, sugarcane, rice, and sorghum due to FAW are approximately US$13 billion every year in SSA [12]. However, earlier studies have established that FAW is a complex pest belonging to the Noctuidae family of insects whose developmental stages and timing are irregular across seasons, hence the complexity of its distribution mechanism [13]. Additionally, the FAW life cycle length exhibits unique variations in development across seasons i.e., in summer it is about a month while in spring and autumn it takes approximately two months, and three months during winter [14,15]. Furthermore, FAW populations increase rapidly under warm, humid conditions with moderate rainfall and an optimal temperature range between 11 °C and 30 °C with the female moth laying approximately 1000 eggs in its lifetime in masses varying from 50 to 200 eggs at each lay [14]. Moreover, FAW is a migratory pest with the potential to cover long flying distances of approximately 1000 km in its lifetime [11,14]. For instance, Ref. [16], reported that in 30 h, FAW covers a distance of ∼1600 km from the southern state of Mississippi in the United State of America (USA) to southern Canada. Also, the FAW is a polyphagous pest that feeds on about 350 plants with a huge preference for species of grasses [[17], [18], [19]]. These phenomena enhance the complexity of understanding the FAW biogeographic ecologies in the different regions as the dynamics change according to the geographical location.

This generates challenges to customize strategies for FAW management using contemporary methods for pest control such as integrated pest management (IPM) [12,20]. Thus, accurate on-farm scale mechanisms are essential to inhibit the invasion of new areas within the first few days of detection [12,21,22]. However, these control technologies require spatially explicit landscape-scale information that shows potentially suitable sites for FAW occurrence and establishment to enable localized interventions [23,24]. Regrettably, landscape-scale necessary information on pest occurrence, spatial infestation extents, and abundances remain rudimentary in SSA, particularly in eastern Africa, which is the hub of cereal crop production [12,25].

Earlier studies have concentrated on the bio-ecological insights of the FAW i.e., the ecologically preferred conditions, migratory behavior, morphology, and biological development [12,26,27]. Again, more literature has focused on FAW surveillance and monitoring [28], its potential impacts on crop production [8,14,25], the potential management strategies including farming systems [15], modeling the potential pest population growth [23] and detecting crop damage caused by the pest using remotely sensed data [29,30]. Besides, most research that have predicted FAW habitat suitability have used the maximum entropy (MaxEnt) and CLIMEX ecological niche modeling (ENM) approaches at global or continental scales [22,[31], [32], [33]]. However, there is still a need to predict the spatial distribution and establishment of FAW at a more localized landscape-scale mainly targeting data deficient regions such as eastern Africa. Moreover, The MaxEnt is the widely used algorithm to simulate suitable habitats for a diverse number of species in different taxa by many earlier studies worldwide (e.g., Refs. [22,[34], [35], [36]]. Specifically, MaxEnt is consistently superior in its predictive capacity, adaptability, practicality, and robustness as supported by the over 2000 ecological studies that have used MaxEnt since 2006 [37]. Also, the MaxEnt algorithm was selected in most of these studies because it can comparatively work with a small sample size of presence-only observations unlike other algorithms [38]. Furthermore, previous studies have mostly utilized climatic, edaphic, and irrigation variables to predict FAW habitat suitability without testing the landscape structure effect and relevance on the spatial distribution and establishment of the pest. Landscape structure includes natural or semi-natural habitats that could be conducive to or hinder the insect pests (e.g., FAW) build-up [12]. For instance, hedges and grasses neighboring croplands can provide pathways for FAW in an agroecosystem and probably act as secondary hosts for the pest, depending on the specific plant species compositions [31]. Moreover, land surface temperature (LST) can vary as a function of landscape structure and positively or negatively affects the occurrence and establishment of crop pests like FAW [39]. Hence, establishing the potential risk posed by FAW involves setting up ecologically significant and uncorrelated explanatory variables [40,41]. To include less correlated variables in species distribution models (SDM), an elimination procedure of some of the correlated variables should be conducted to reduce the chances of model overfitting and variable inflation [42]. Commonly, bioclimatic variables such as the ones obtained from the WorldClim [43] are highly correlated because they are all derived from similar data [44]. These bioclimatic variables are widely used in habitat suitability models like the one employed in this study.

Therefore, the present study aimed to predict the occurrence and establishment of FAW in five east African countries viz., Kenya, Tanzania, Rwanda, Uganda, and Ethiopia using a SDM i.e., MaxEnt and bioclimatic, LST, wind speed, elevation, and landscape structure metrics (i.e., LULC and maize harvested area) as explanatory variables. Also, the study assessed the relevance and influence of each of the studied variables on the FAW spatial distribution and establishment. We hypothesized that FAW invaded almost the entire SSA since it was first reported in 2016, hence the suitable niches (habitats) that can be conducive to its build-up and establishment should be predicted. Furthermore, the impact of simulated future climate change scenarios for the years 2030 and 2050 on FAW spatial distribution patterns and pest establishment using the medium shared socio-economic pathway (SSP2-4.5) and the highest (SSP5-8.5) emission scenarios were evaluated. The SSP concept was coined by the intergovernmental panel on climate change (IPCC) using the radiative forcing level of 4.5, and 8.5 W m^−2^ [45]. Specifically, our study is unique and innovative as it predicts FAW establishment risks at a landscape scale in five east African countries using relevant climatic and landscape structure variables (metrics) and a machine learning SDM. In addition to the 19 bioclimatic variables [43], we used wind speed, solar radiation, LULC and the main FAW host crop area (i.e., maize) in eastern Africa as the most relevant explanatory variables.

## 2 Study area

**Fig. 1:**
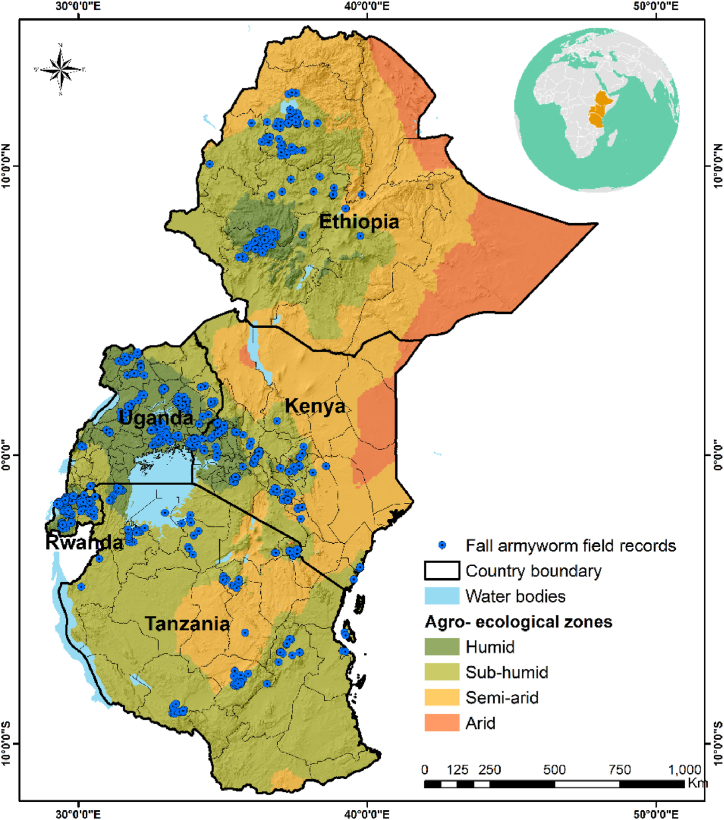
The distribution of fall armyworm (FAW) occurrence data in eastern Africa. The blue dots show the areas where the occurrence of FAW was recorded. These data are overlayed on the agro-ecological zone layer of the five countries.

The study was conducted in Kenya, Tanzania, Rwanda, Uganda, and Ethiopia (Fig. 1). These countries lie between latitudes 16° N and 11° S and longitudes 30° E and 48° E. In general, all these countries are characterized by various climatic conditions (arid to tropical monsoon) and span across different agro-ecological regions. Temperatures are generally high throughout the year in the highlands of Ethiopia and Kenya, while Tanzania experiences relatively cooler temperatures. The mean annual temperature in the region is highly variable with a range of −5 °C–31 °C, and an average value of 22.9 °C [43]. The high temperature variability is mainly due to various climate types in the region such as equatorial, moist and dry tropical and semi-arid and arid climates. In general, the region became relatively warmer by 0.7–1 °C between the years 1973–2013 [46]. Likewise, the altitude in East Africa varies between 1082 and 5780 m (above sea level). In the period from June to August, December to February, and October to April, the Northern and Southern regions of eastern Africa receive much of the rains whilst the areas around the equator experience two rainfall seasons, from March to May (the long rainy season) and from October to December (the short rainy season), respectively [47]. The region records an average annual rainfall range of approximately 150 mm in semi-arid and arid areas to around 2000 mm in the highlands. The rainfall in the region is enough to support sustainable agriculture, despite the dynamism in the climatic conditions that adversely impact crop productivity.

The common cropping systems in these eastern African countries range from monocropping, mixed cropping, and crop rotation to sequential cropping systems [48]. Maize is the staple food crop in these countries, thus is the most planted crop and is often planted as an intercropping system with leguminous crops such as bean, pigeon pea, cowpea, groundnut, and soybean. Intercropping and other crop husbandry practices that have been widely adopted in eastern Africa can alleviate pest infestation and build-up such as FAW. Unfortunately, most of the populace in eastern Africa affected by the FAW are the millions of small-scale farmers who are mostly reliant on an agriculture-based economy for their livelihood and sustenance. Therefore, there is a need to monitor FAW and find sustainable solutions to manage the pest at scale.

## 3 Methodology

### 3.1 Fall armyworm (FAW) occurrence data

The FAW occurrence (presence-only locations) dataset comprised 2621 reference points that were obtained from three secondary sources i.e., the Food and Agriculture Organization (FAO) of the United Nations, the Center for Agriculture and Bioscience (CABI), and the Global Biodiversity Facility (GBIF). The long-term FAW field data collected through the FAO monitoring and collection initiative using the FAW monitoring and early warning system (FAMEWS: n = 2531) mobile application was used as the core reference data source. The data are available for 2018, 2019, and 2020 and freely downloadable on the FAO platform (http://www.fao.org/fall-armyworm/en/). Again, additional data (n = 80) were acquired from the GBIF (https://www.gbif.org), an online database that hosts over a billion global biological records. The third data source, i.e., CABI (n = 10), provides one of the world's most comprehensive datasets on crop pests with over 27,000 datasheets already available in their crop protection and compendium (CPC) database (https://www.cabi.org/cpc/). The data from these three sources were standardized and cleaned to remove samples of the same coordinates and the ones without coordinates. Furthermore, a single sample was retained within a 1 km × 1 km unit to reduce sampling bias and to meet the spatial resolution of the environmental variables following [49]. The retained samples (n = 1190) were validated for location accuracy using Google Earth (https://www.google.com/earth/). These retained FAW presence-only reference points were used as independent response data in the MaxEnt modeling approach as well as for producing the sampling bias file [49,50].

### 3.2 Dependent explanatory variables

The dependent explanatory variables that were used in this study included bioclimatic, elevation, LULC, LST, wind speed, maize harvested area, and solar radiation. These variables were obtained from different sources at differing spatial and temporal resolutions. This step is critical as these spatial and temporal dynamics determine a dataset's suitability for use in modeling as their inclusion often influences the observed patterns of the analysis [51]. However, the objectives of a study ultimately determine the appropriateness of the spatial and temporal resolutions of the explanatory variables, hence there is no absolute best resolution for all modeling experiments [52]. In this study, the variables' spatial resolution varied from a pixel size of approximately 20 m × 20 m (for LULC) to 10 km × 10 km (for maize harvested area). Such differences inhibit the integration of multi-resolution variables in the model experiments, particularly for the MaxEn approach. Therefore, in this study, the spatial resolution differences among the dependent explanatory variables were counteracted by matching and resampling all the datasets to the bioclimatic variables (1 km × 1 km grid cells), and all datasets were matched to the boundary of the study area (the five counties in eastern Africa). The ‘raster’ package [53] in R software [54] was used for all resampling and re-sizing of the study area. This procedure was performed using the ‘crop’ and ‘mask’ functions.

Furthermore, a total of 19 bioclimatic variables together with wind speed and solar radiation were acquired from the Worldclim data portal (https://worldclim.org/data/index.html). These variables were initially used together with the other explanatory variables to select the most optimum variables to use in the subsequent modeling experiment. The bioclimatic variables from the Worldclim data portal comprise gridded spatial climate parameters at a resolution of 1 km × 1 km comprising annual trends, seasonality, precipitation of the wet and dry quarters, and extreme climatic events generated from long-term average climate data records that ranged temporally from 1970 to 2000 [43]. Again, the LULC data at 20 m × 20 m pixel size was obtained from the European space agency (www.esa-landcover-cci.org), while the elevation of 30 m × 30 m pixel size was sourced from the shutter radar topographic (SRTM) mission (https://www.usgs.gov/). Day and night Terra-satellite-based LST observations were acquired from the MODIS instrument (product MOD11) (https://modis.gsfc.nasa.gov/data/dataprod/mod11.php). As previously mentioned, LULC, LST, and elevation will likely affect the incidence and spread of FAW by modifying precipitation patterns and amounts, temperature dynamics, the type of vegetation, cropping patterns, and the direction, angle, and intensity of the sun on the Earth's surface [55,56].

Additionally, to ensure that the models were run within the maize cropping area, which is the main FAW host crop in eastern Africa, a 10 km × 10 km gridded dataset on maize harvested area was sourced from the spatial production allocation model (MapSPAM) data center by HarvestChoice (https://www.mapspam.info/data/), and used in our model experiment.

### 3.3 Dependent explanatory variable selection

A two-stage variable removal criterion utilizing the cluster analysis of the 19 bioclimatic variables and their variance inflation factor (VIF) was performed to facilitate the decision of variable retention or exclusion in the model. The first step for variable selection involved the exploration of the variable clusters (Supp. 1) using Pearson's correlation coefficient and the cluster tree provided in the ‘virtual species’ package [57] in R [54]. Of the nineteen bioclimatic variables, only twelve were selected from this procedure. The excluded variables include Bio7, Bio8, Bio11, Bio13, Bio14, Bio18, and Bio19, and a cutoff of |r| = 0.7 was used [58]. The twelve selected bioclimatic variables were also selected due to their ecological significance in predicting the suitability of other insect pests, including the FAW as evidenced by previous studies [59].

The bioclimatic variables obtained from the cluster analysis step were combined with other six explanatory variables (Table 1) and were further tested for correlation using the VIF approach provided in the “usdm” package [60] in R. The “vifcor” function was used to eliminate variables with the highest VIF (VIF ≥10) [23,42,60]. The VIF iteratively performs multiple linear regression analysis and detects multicollinearity by regressing each explanatory variable against the other variables [61]. Visualization and analysis of the correlation matrix were then used to further evaluate the variables (Supp. 2). A total of eighteen variables were retained for the MaxEnt modeling experiment to simulate the spatial distribution of FAW.

**Table 1:** Dependent explanatory variables considered in the predictive modeling of fall armyworm (FAW) spatial distribution in five countries in eastern Africa.

Variable	Description	Units
Bio1	Annual mean temperature	°C
Bio2	Mean diurnal range (mean of monthly (max temp - min temp))	°C
Bio3	Isothermality (BIO2/BIO7) ( × 100)	NU^a^
Bio4	Temperature seasonality (standard deviation × 100)	°C
Bio5	Maximum temperature of warmest month	°C
Bio6	Minimum temperature of coldest month	°C
Bio7	Temperature annual range (BIO5-BIO6)	°C
Bio8	Mean temperature of wettest quarter	°C
Bio9	Mean temperature of driest quarter	°C
Bio10	Mean temperature of warmest quarter	°C
Bio11	Mean temperature of coldest quarter	°C
Bio12	Annual precipitation	mm
Bio13	Precipitation of wettest month	mm
Bio14	Precipitation of driest month	mm
Bio15	Precipitation seasonality (Coefficient of Variation)	NU^a^
Bio16	Precipitation of wettest quarter	mm
Bio17	Precipitation of driest quarter	mm
Bio18	Precipitation of warmest quarter	mm
Bio19	Precipitation of coldest quarter	mm
Land surface temperature	Surface temperature and emissivity	K
Land use/Land cover	Land cover classes in the area	NU^a^
Elevation	The terrain of the land surface	m
Wind speed	Speed of the wind	m s^−1^
Solar radiation	Top-of-atmosphere incident solar radiation	kJ m^−2^ day^−1^
Maize area	Maize harvested area	ha

a NU = no unit.

### 3.4 Maximum entropy (MaxEnt) model settings and accuracy assessment

The MaxEnt model (version 3.4.1) [62] was used to simulate the establishment areas for FAW in five countries in East Africa. The optimal settings (tuning and parameter) for the MaxEnt model used in this study were derived from the ‘ENMevaluate’ function in the package ‘ENMeval’ [63] available in R [54]. The ‘ENMevaluate’ function has been recommended by earlier studies as it calculates numerous measures and parameters that aid in choosing the most appropriate model settings that ensure a balance between the goodness-of-fit and model complexity. This is achieved using the actual presence-only data points of the target species [38,63,64]. The most optimum modeling parameters established from the “ENMeval” for the FAW were: hinge: 0.5, beta-multiplier: 5.0, categorical: 0.3, threshold: 1.6, linear/quadratic/product: 0.2, clamping, extrapolate, fade with clamping and the multivariate environmental similarity surface (MESS) analysis. The MESS analysis computes the uncertainty of the prediction by estimating the differences of each pixel in the projected region to a set of reference points [65], in our case into the future where the occurrence validation data can not be obtained.

We utilized the ‘kde2d’ function of the ‘MASS’ package [66] which estimates the kernel density using the ‘block’ sampling method in R [54] for sampling bias reduction. The ‘kde2d’ function achieves bias reduction by creating a two-dimensional kernel density estimate using the longitude and latitude from the reference points [66]. The generated bias file was then systematically included in the MaxEnt model to facilitate the generation of background data with similar bias as the occurrence points.

Additionally, the sub-sampling method was used together with the above-mentioned setting parameters to develop three replicates for the MaxEnt model. These were then averaged to define the best possible suitability and performance of the model. Following earlier studies, the approach of using 70% (n = 833) of the FAW occurrence points for training and 30% (n = 357) for testing the goodness of fit of the model was adopted in the current study. The significance of each explanatory variable was evaluated using the permutation importance, percentage contribution, and the Jackknife test [62]. Herein, we reported the area under the curve (AUC) of the present climatic conditions, because there are no future FAW occurrence points to corroborate our forecasting. Thus, we assumed that if the model is robust and accurate using the present data, it would replicate the same precision in future forecastings [40].

Graphic maps of the prediction were generated using the MaxEnt model to illustrate the FAW spatial distribution patterns (established habitat) with values ranging from 0 (unsuitable) to 1 (most suitable). Five suitability categories of FAW establishment, i.e., very low (0–0.1), low (0.2–0.3), moderate (0.4–0.5), high (0.6–0.7) and very high (0.8–1) were used. The FAW establishment areas under current and future (2030 and 2050) climatic conditions for these five suitability categories were thereof calculated. The abstract workflow adopted in this study is shown in Supp. 3.

## 4 Results

### 4.1 MaxEnt model evaluation

The replicated ‘testing’ and ‘training’ MaxEnt models to predict FAW habitat suitability indicated a balanced goodness-of-fit and complexity. Specifically, results revealed that the prediction of FAW establishment areas in eastern Africa using the MaxEnt model were based on the model strength and true performance (AUC = 0.87), and not a random chance (Supp. 4). The high AUC value obtained from the replicated MaxEnt models encourages the application of the model for examining FAW establishment areas under current and future climatic conditions.

### 4.2 Analysis of variable importance and contributions

The importance of bioclimatic, LULC, and maize area explanatory variables that accounted for the level of established FAW habitats is shown in Table 2 and Supp. 5. The table shows that the most contributing variable to the model performance was LULC, which contributed 30%. This was followed by Bio17 (precipitation of driest quarter), Bio3 (isothermality), maize harvested area, and solar radiation, which all contributed more than 7% each. The five most contributing variables had a combined contribution of 67.7% leaving the remaining 32.3% to the rest of the 13 variables selected to predict FAW occurrence (Table 2). The lowest percentage contribution to the model was observed for Bio6 (minimum temperature of the coldest month). While the analysis of percentage contribution demonstrated that LULC had the highest relevance, the model permutation importance showed that Bio12 (annual precipitation) had the highest importance followed by LULC, solar radiation, and maize area. The results showed that essentially LULC, maize harvested area, solar radiation, precipitation, and temperature are fundamental in defining the spatial occurrence of FAW and its established habitat.

**Table 2:** The average permutation importance and percentage contribution of the explanatory variables used for predicting fall armyworm (FAW) establishment and habitat suitability in eastern Africa.

Variable	% contribution	Permutation importance
LULC	30.0	20.4
Bio17	12.1	03.4
Bio 3	09.4	01.7
Maize area	08.8	10.1
Solar radiation	07.4	11.1
Elevation	06.6	04.6
Bio15	06.1	07.2
Bio12	03.9	27.9
Bio5	03.5	00.4
Bio2	02.6	02.0
Land surface temperature	02.4	00.5
Bio16	02.0	00.6
Bio9	01.9	02.9
Bio4	01.1	00.5
Bio1	00.8	00.8
Bio10	00.7	00.2
Wind speed	00.5	01.4
Bio6	00.2	04.2

The results showed that all the variables had relatively high AUC values (Supp. 5) when used in isolation (AUC >0.55). However, three variables particularly stand out i.e., maize harvested area, LULC, and Bio12 (annual precipitation) as the most dominant variables, which can be used in isolation and still provide an accurate model (AUC >0.7). However, only maize harvested area and LULC showed the greatest reduction in the model accuracy if they were to be excluded from the model (±0.2 reduction in AUC). The bioclimatic factors also showed high levels of importance with Bio17 (precipitation of driest quarter), Bio12 (annual precipitation), and Bio3 (isothermality) having high contributions to the AUC in determining FAW establishment in eastern Africa. The variables with the least AUC when used in isolation were LST, Bio2, Bio16, wind speed, and solar radiation. Although solar radiation showed low AUC values using the Jackknife test, the percentage contribution and permutation importance (Table 2) proved that it is an important variable in determining the established habitat for FAW in East Africa.

The curves in Supp. 6 demonstrate the minimal influence of altering a single variable on the performance of the MaxEnt model. They showed how the FAW simulated established habitat varies because of a specific variable, while maintaining all the other variables at their mean sample value. Supp. 6 demonstrates the response of the six most contributing variables (i.e., LULC, maize harvest area, elevation, Bio3, Bio17, and solar radiation) to the MaxEnt model performance. The response of the LULC variable showed that built-up area (class number 8) and cropland (class number 4) were the most important classes for determining the established habitat or the occurrence of FAW in eastern Africa (Supp. 6a). Furthermore, the results showed that as the area covered by maize increased the area of FAW establishment also increased, with maize areas of 4000–16000 m^2^ having suitability scores >0.75 (Supp. 6b). Similarly, a broad range of elevations were suitable for FAW establishment, and the level of pest occurrence generally increased with increasing altitude (Supp. 6c). The response obtained from Bio3 showed that FAW occurrence could take place across a range of isothermality with the level of occurrence steadily increasing at an isothermality range of 45–87 (Supp. 6d). This indicates that large fluctuations in mean monthly temperatures relative to annual temperatures are conducive conditions for FAW establishment. On the other hand, the results demonstrated that wetter conditions and high solar radiation reduced the level of occurrence of FAW (Supp. 6e and 6f, respectively).

### 4.3 Fall armyworm (FAW) establishment risk areas under current and future climate conditions

**Fig. 2:**
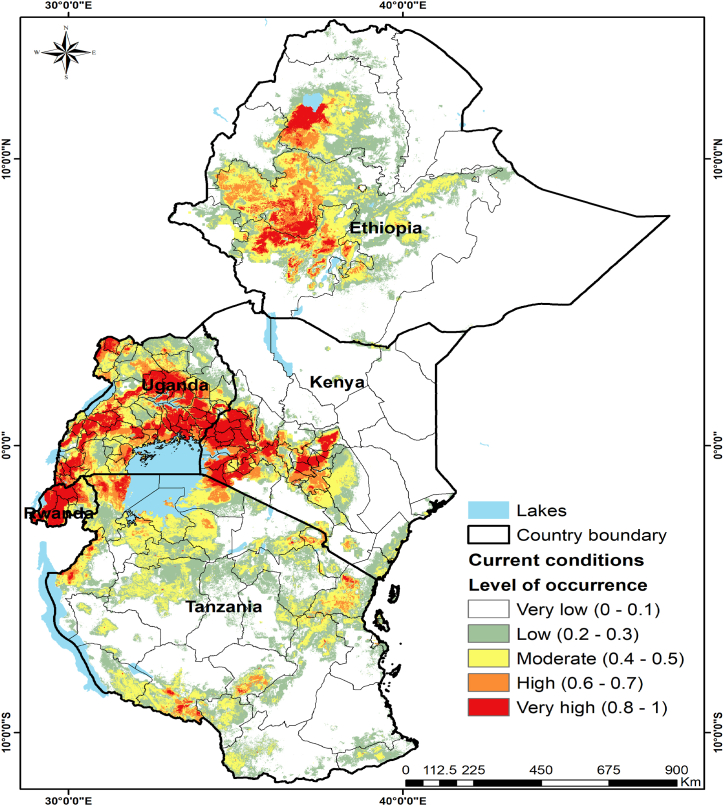
Fall armyworm (FAW) level of occurrence (establishment risk areas) in eastern Africa under current climate conditions. The red color shows the highest probability of occurrence and establishment, while the green color shows the lowest probability. The yellow and orange colors show moderate and high FAW levels of occurrence, respectively.

**Fig. 3:**
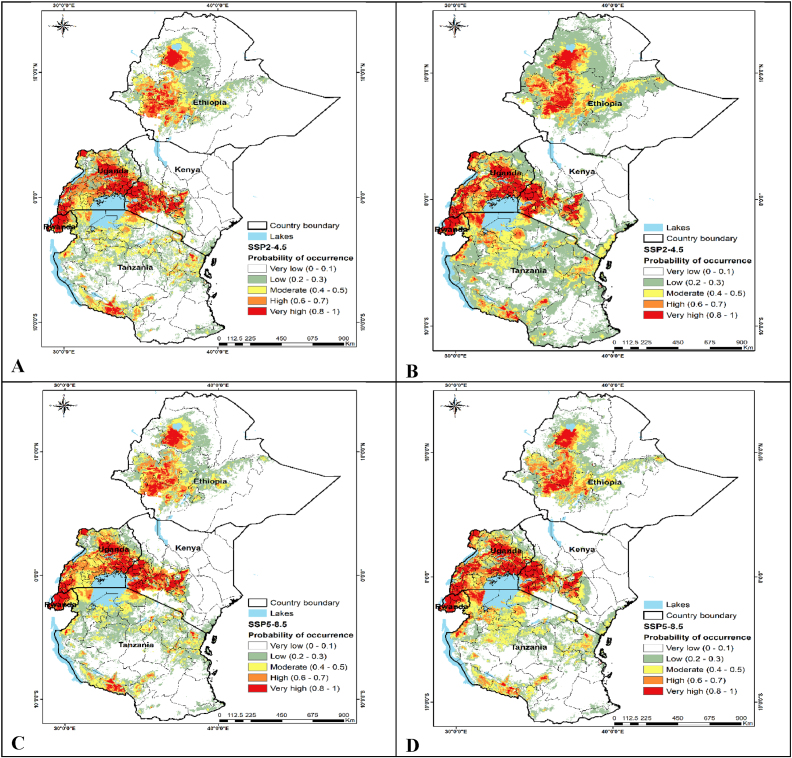
Fall armyworm (FAW) level of occurrence (establishment risk areas) in eastern Africa under future climate scenario using the shared socio-economic pathways (SSP2-4.5) of 2030 (A), and 2050 (B); and SSP5-8.5 of 2030 (C) and 2050 (D). The red color shows the highest level of occurrence and established habitat while the green color shows the lowest. The yellow and orange colors show moderate and high FAW levels of occurrence, respectively.

Notably, most sites in eastern Africa where FAW establishment risk is high are within the semi-humid to humid agroclimatic zones, where maize is the most grown field crop (Fig. 2). In contrast, arid to semi-arid climates exhibited the lowest FAW establishment risk. The areas of high establishment level for the pest are mostly in central Ethiopia along the highlands, the Lake Victoria regions of Kenya, Uganda, and Tanzania, and also within the central, western, and eastern regions of Rwanda. Although the locations and patterns of the future (2030 and 2050) potential FAW risk areas that are demonstrated in Fig. (3) look relatively similar to those of the current scenario, our models' results suggested that the level of establishment and magnitude of FAW risk would generally increase under the two tested climate change scenarios (SSP2-4.5 and SSP5-8.5). This change in the magnitude of the pest establishment risk is also demonstrated in Table 3. This holds, particularly for the maize growing regions in the five study countries.

**Table 3:** The predicted coverage (km^2^) and percentages of the established area of fall armyworm (FAW) risk (level of occurrence) in eastern Africa using current and future shared socio-economic pathways i.e., SSP2-4.5 and SSP5-8.5 climate scenarios for the years 2030 and 2050.

Level of occurrence	Area (km^2^) under current climate conditions	% of suitable area	Area (km^2^) under future SSP2- 4.5 climate scenario in 2030	% of suitable area	Area (km^2^) under future SSP2- 4.5 climate scenario in 2050	% of suitable area	Area (km^2^) under future SSP5- 8.5 climate scenario in 2030	% of suitable area	Area (km^2^) under future SSP5- 8.5 climate scenario in 2050	% of suitable area
Very low	934,261	41	867,192	38	637,720	28	834,461	37	712,427	31
Low	716,413	31	759,143	33	803,309	35	786,449	34	784,764	34
Moderate	304,701	13	311,712	14	403,755	18	311,453	14	381,527	17
High	159,213	07	165,670	07	240,612	11	168,266	07	267,267	12
Very high	168,877	7	179,746	08	200,110	9	184,879	08	139,521	6
Total	2,283,465	100	2,283,465	100	2,285,506	100	2,285,506	100	2,285,506	100

Table 3 shows area coverage and percentage of FAW established habitat (level of occurrence) in the five east African countries. In general, the results showed that in the future there will be an increase in the moderate, high, and very high FAW occurrence and establishment areas, with the highest occurrence area for these three classes (38% of the area) predicted under the SSP2-4.5 climate scenario in 2050. In 2030, the total area of the three above mentioned suitability clases was 29% of the total area for each of the two climate scenarios. Interestingly, the moderate to very high suitable areas for FAW establishment were progressively increased over time (current, 2030 and 2050), except the slight decrease in area of the very high class under SSP5-8.5 climate scenario of 2050 (Table 3). Moreover, in the future the suitable FAW establishment areas will increase to about 29% under each of climate scenario of 2030, and to 38% and 38% under SSP2-4.5 and SSP5-8.5 climtate scenarios of 2050, respectively as opposed to the one under the current climate conditions (27% of the area). On the contrary, there will be a decrease in the least suitable (very low class) areas of FAW establishment under the future climate change scenarios in comparison to the current climate conditions. This could indicate that a large proportion of the least suitable areas of establishment shall develop a susceptibility to FAW invasion under future climate change scenarios. In other words, the currently very low FAW established areas would shift to the low, moderate, high, and very high levels of pest establishment as a result of climate change.

## 5 Discussion

The study presents a SDM model that predicted the geographical distribution of the potential FAW establishment area in five east African countries. The results showed that FAW suitable establishment localities are distributed across a wide range of agro-ecologies in eastern Africa based on the investigated climate scenarios and landscape structure (i.e., LULC). This is supported by earlier studies, which have predicted that tropical and subtropical climates such as those experienced in eastern Africa are the most fit for the all-year-round invasion of FAW compared to temperate regions, which are likely unsuitable or at low risk of seasonal invasions [32] since FAW does not diapause [67]. Despite the fact that FAW can migrate to uninhabitable areas during its population booms, it is not necessary the conditions in these areas are suitable for the pest to survive. In such a case, the pest cannot complete its cycle even for one generation. Therefore, it is expected that the FAW will continue to invade new areas and cause considerable production losses to crops like maize, but not necessarily to build-up and establish unless the conditions are suitable. We recommend that FAW population build-up and spread should efficiently be monitored and managed to reduce such crop losses [68]. Thus, our study becomes extremely critical and timely as it predicts the spatial variability of FAW occurrence on a wider scale, leading to coordinated regional and inter-country planning and collaboration to better manage the pest. Particularly, this requires that policymakers and stakeholders in East Africa should strengthen their exchange and cooperation to jointly slow the further spread of FAW in the region.

Predicting establishment areas for highly mobile pest species such as the FAW is a demanding and complex task that involves a selection of very relevant explanatory variables, which best mimic the environmental conditions of the pest. Also, modeling the spatial distribution of such a highly mobile pest requires multi-year occurrence data at a landscape scale. In the present study, we utilized FAW occurrence observations that were collected over three years (2018–2020). This period coincided with the peak of FAW invasion in Africa [20]. Furthermore, in our modeling experiment, we included the most preferred FAW host crop i.e., maize [31], which provides food and nutrition security to most of the populace in sub-Saharan Africa including eastern Africa [68]. Moreover, we tested the influence of long-term bioclimatic, and 3-year elevation, solar radiation, and wind speed, as well as LULC variables/indicators on predicting FAW establishment. Our multi-year FAW observations and long-term average bioclimatic and climatic variables reinforce the reliability and robustness of our modeling approach as multi-date observations are usually more valuable than snapshot observations. This reduces the expected intermediate (year-to-year) variability and noise in the data, hence enhancing the model performance [69]. Notwithstanding, we did not use near-real-time bioclimatic variables that might have better explained the year-to-year spatial variability in the FAW occurrence data points. On the other hand, multi-year biological data at larger scales (e.g., region or continent-wide) are commonly collected through citizen science approaches, which allow public engagement in data collection and sharing to improve scientific knowledge [70]. However, citizen science observations could be spatially biased and lack information on survey efforts as the observers may tend to oversample accessible areas (e.g., maize fields next to main roads). This was countered in the present study by creating a sampling bias file in the MaxEnt modeling experiment [50,71,72].

The above-mentioned explanatory variables proved very relevant for predicting FAW establishment areas under current and future climate regimes. The relevance of these explanatory variables was demonstrated by their contribution to the predictive model performance and the relatively accurately (AUC = 0.87) predicted FAW establishment maps. It is interesting to note that our study was the first attempt to investigate the influence of localized LULC, wind speed, and solar radiation on the distribution and risk of FAW.

The LULC in general, and maize harvested area, in particular, contributed the most to the performance of our FAW predictive model. This is in agreement with other study findings that landscape structure influences insect pest occurrence and abundance through the “scale dependence” concept [73]. This means that FAW influence/presence and interactions at a field scale, for instance, could be affected by spatially explicit landscape patterns, like the proportion coverage of certain LULC classes (e.g., grassland, bushland, etc.) [74]. In particular, surrounding and specific LULC classes could form secondary hosts for FAW, specifically during cropping season breaks [32]. Also, landscape structure could affect the prevailing bioclimatic or micro-climatic conditions that influence the development, survival, and abundance of the pest at the field scale [74]. The highest contribution of maize harvested area to FAW predictive model performance is expected since maize is the pest's main host crop and the most grown crop in the study area [20,75]. It is known that FAW is a polyphagous pest, which feeds on over 350 plants although maize is among its most preferred hosts [18,76]. The high importance of LULC and maize harvested area in our MaxEnt model results, therefore, indicated that if the secondary host plants are available at a landscape scale, FAW may remain active throughout the year even when the maize crop at a field scale is seasonally unavailable. Hence, the pest should also be managed in the surroundings of the fields of the main host crop (e.g., maize), particularly during cropping season breaks. However, in eastern Africa there are two maize growing seasons supported by the ‘short’ and ‘long’ rains seasons, making the management of the pest more challenging. Specifically, this can lead to FAW carryover from primary host crops to secondary host plants, and from one season to another enhancing the pest propagation. The high influence of the build-up class (urban or rural settlement clusters) on our FAW habitat suitability could be due to the association of built-up areas with intensive maize farming areas in eastern Africa, particularly in small-scale farming areas. Consequently, future studies should include maize planting period in FAW establishment prediction experiments, should a geospatial (gridded) maize planting time dataset is available. This will allow the prediction of FAW actual hazard level in east Africa as a function of maize planting season.

The second most contributing variables to our MaxEnt model were temperature, precipitation of the driest quarter, and solar radiation. In general, studies have reported that climate variability considerably affects the distribution and abundance of insect pests such as FAW [33,77,78]. This was demonstrated by the response curves (Supp. 6) and how an increment in temperature increased the FAW potential invasion scores (habitat suitability). These climate responses could have triggered the massive FAW migration from its endemic region in South America. Specifically, temperature-based variables were described by previous studies as a vital aspect of the insects' development cycle, survival, and abundance [23,79]. Although temperature variables substantially affect insect abundance [79], they do not determine migration patterns on their own. Insect migration patterns are also highly sensitive to seasonal variations and interactions in other environmental and bioclimatic variables occurring over the years [80,81]. Thus, it was clear in this study that FAW establishment was more sensitive to isothermality (Bio3). This, however, complicates the understanding of the FAW spatial distribution as temperature and precipitation across the seasons of the year influence the FAW potential establishment risk [82]. The influence of the precipitation on the predictive model could be twofold; (i) indirectly through boosting the vegetative growth and development of the FAW primary and secondary host crops/plants , which results in FAW population build-up [83], and (ii) directly through its influence on the pest occurrence, its survival and development [31]. However, FAW larvae can be washed away by raindrops. In general, our study shows that the spatial occurrence of FAW largely depends on optimum interactions of a wide range of explanatory variables. But their mechanisms of interaction are nonetheless currently vague [83,84]. These variables vary from field size and geographical location to the cropping calendar and the FAW host plants as well as the pest management technologies adopted by the farmers [25]. Therefore, there is a need for a holistic FAW early warning and monitoring system in Africa.

Similarly, other studies have carried out modeling experiments to predict the spatial distribution of FAW at various scales since it was first reported in Africa in 2016 [21,22,31,33,82,85]. In particular, these studies have employed SDMs to provide a quick understanding of the potential spatial dynamics of the pest. However, these studies mainly aimed at predicting FAW suitable ranges and expansion at often a global or continental level. Therefore, their results could not explicitly inform FAW spatial distribution at a regional or local scale due to spatial heterogeneity within the landscape, and a multitude of factors that influence the pest occurrence across different agro-ecological regions including adaptation [86]. Thus, the results of the present study, which showed the FAW spatial distribution and establishment at a regional scale are useful to pinpoint intervention priority locations to manage the pest within a specified, i.e., similar environmental conditions and landscape setup, geographical space.

Notably, the results indicated that high to very high-risk FAW establishment areas are mostly located in areas of relatively higher rainfall (i.e., semi-humid and humid agro-ecological zones in eastern Africa). Moreover, an in-depth analysis of the spatial distribution of FAW in eastern Africa showed that FAW has adapted to the conditions of relatively higher temperatures and low windspeed. This emphasizes the relevance and importance of precipitation regimes, and temperature and wind speed profiles in predicting FAW distribution. The areas of high establishment for the pest are mostly in central Ethiopia along the highlands, the Lake Victoria regions of Kenya, Uganda, and Tanzania, and also within the central, western and eastern regions of Rwanda. Also, in the areas that were predicted to have high and very high scores of FAW establishment (Fig. 2), maize is the most grown crop. Again, maize is the main FAW host crop in the study area. Nevertheless, the results showed that only 14% of the study area had high to very high FAW suitability scores under the current climatic conditions (Table 3). Since maize harvested area was among the most contributing variable to our MaxEnt model, the low area coverage of these suitability scores was expected as the crop is mainly grown by smallholders in the five study countries [87]. Also, we did not use real-time climatic variables that could have timely explained the FAW risk of establishment. In contrast, the regions where the climate is arid to semi-arid exhibited the lowest FAW establishment risk. Furthermore, our study predicts that future FAW risk of the establishment will increase as a result of climate change under both SSP2-4.5 and SSP5-8.5 scenarios. This is in accordance with other studies' findings that climate change increases the risk of FAW [32] and other maize invasive insect pests like stemborers [88].

The outputs obtained in this study were FAW predictive analyses that might somewhat be different from the actual situation in eastern Africa. Also, the accuracy of our predictive models can be hindered by the expected inherent limitations and uncertainties of the used SDM. Thus, the current predicted state of the FAW distribution needs to be further verified by comparing its predictions with the actual FAW distributions should up-to-date spatially explicit FAW occurrence observations are readily available. Future studies should further investigate whether FAW has adapted to new agro-ecologies that are different from its native conditions. Also, future studies should consider embedding this study's results into a platform that provides near-real-time climate data for the timely prediction of FAW spatial distribution and establishment risk at the farm scale. Besides, future studies should look at estimating FAW growth and population dynamics and eventually develop a decision support system to manage FAW at the farm scale. Such a system allows the estimation of FAW economic injury level that determines the application of control measures to prevent the increment in the pest population to a level that could cause severe crop damage.

## 6 Conclusions

The novelty of this study is that it predicts the establishment risk of the invasive FAW at a landscape scale in five eastern African countries using several relevant explanatory variables that include bioclimatic, climatic (wind speed, solar radiation), landscape structure (LULC), and maize harvested area. Also, our study is the first attempt to utilize wind speed, solar radiation, and the area of the main host crop in predicting the spatial distribution of an invasive insect pest. We demonstrated that LULC, precipitation of driest quarter, isothermality, maize harvested area, and solar radiation are the main ecological indicators for FAW risk of establishment in eastern Africa. Specifically, the results of the present study showed that FAW was already established in several areas in eastern Africa, with an accuracy of prediction (i.e., AUC) of 0.87. Other African countries with similar environmental conditions could also be at risk for FAW establishment. This study demonstrated that climate change will potentially increase the geographical distribution range for FAW. Ecologically, our study implies that FAW will continue building-up and spreading in the suitable areas in the region and SSA in general, particularly given the expansion of maize farming systems in the region. Also, landscape structures, specifically natural landcover classes, could act as secondary hosts for FAW during cropping season breaks, and as corridors for the pest dispersal from one field to another during the season. Overall, our results contribute, as an empirical warning approach, to five countries in eastern Africa, to employ adaptive strategies to manage FAW establishment risk. Thus, preventive measures should be taken to combat the pest spread into areas where they have not yet been reported and/or established. Specifically, phytosanitary strategies, and cultural and biological control measures are necessary for areas that are currently at risk of FAW establishment. Future studies should estimate the abundance (density/population) of FAW at the field scale to develop a forewarning expert system that guides a precise application of IPM technologies in hotspot areas.

## Author contribution statement

Elfatih M. Abdel-Rahman, Emily Kimathi and Bester Tawona Mudereri: Conceived and designed the experiment, performed the experiment, analyzed and interpreted the data, and wrote the paper.

Raphael Mongare: Conceived and designed the experiment, performed the experiment, analyzed and interpreted the data.

Henri E.Z. Tonnang, Sevgan Subramanian and Saliou Niassy: Conceived and designed the experiment and analyzed and interpreted the data.

## Data availability statement

Data associated with this study has been deposited at https://dmmg.icipe.org/dataportal/dataset/a-computational-biogeographic-distribution-of-the-fall-armyworm-in-east-africa.

## Declaration of interest's statement

The authors declare no conflict of interest.

## Additional information

No additional information is available for this paper.

## Declaration of competing interest

The authors declare that they have no known competing financial interests or personal relationships that could have appeared to influence the work reported in this paper.
