# Supplementary material for: Computational biogeographic distribution of the fall armyworm (Spodoptera frugiperda J.E. Smith) moth in eastern Africa
Source: Heliyon. 2023 May 15;9(6):e16144. doi: 10.1016/j.heliyon.2023.e16144 (PMC10230198; doi:10.1016/j.heliyon.2023.e16144)
Supplement: Multimedia component 1 [file mmc1.docx]

**Supplementary materials**


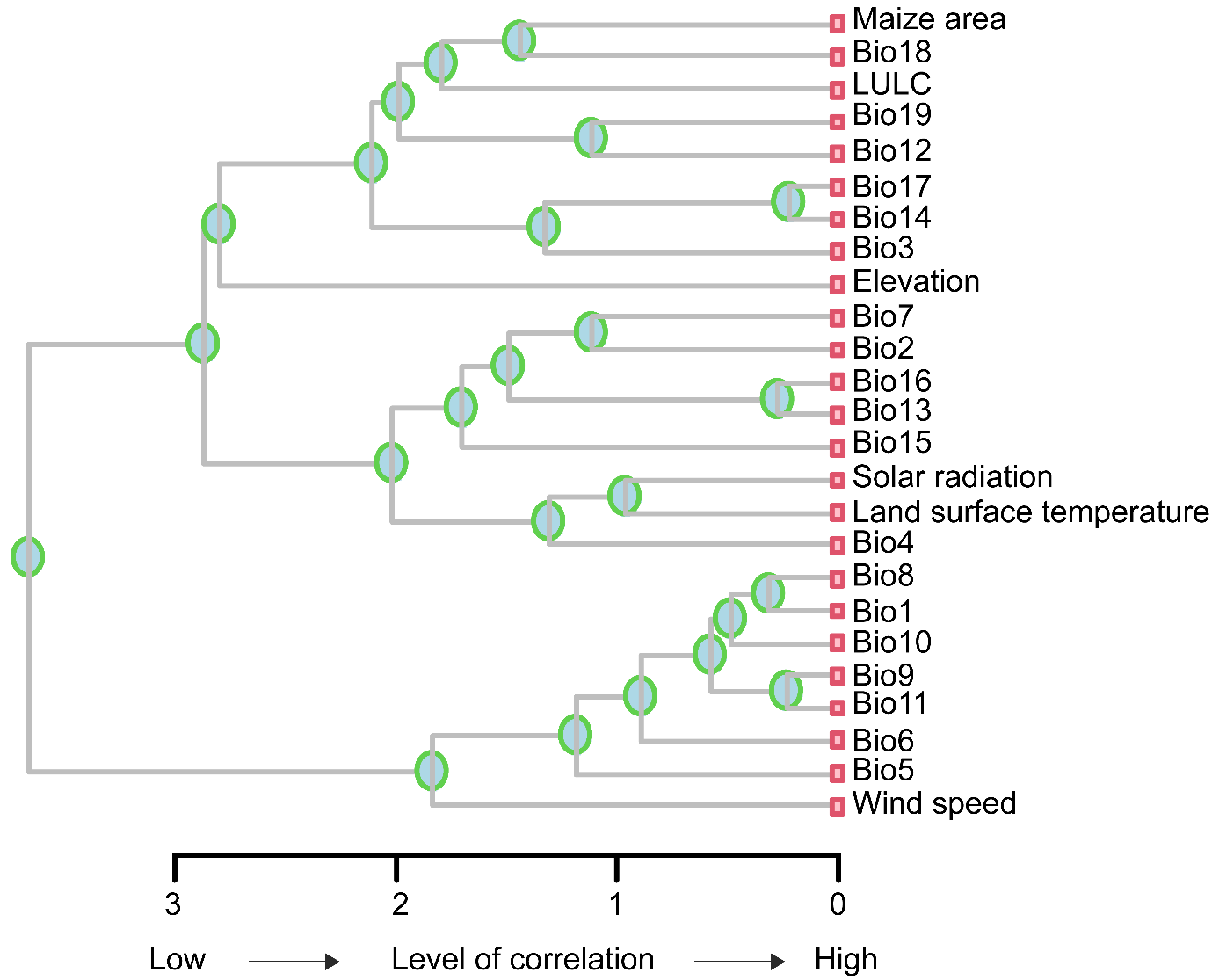


**Supp. 1.** Clustered groups of intercorrelated explanatory variables. The grey connecting lines demonstrate the level of correlation. Variables connected with the grey lines in the same cluster within levels 0 to 1 are highly correlated while those connected with the grey lines between levels 2 and 3 have low correlations, and levels between 1 and 2 are termed intermediate with moderate correlation.


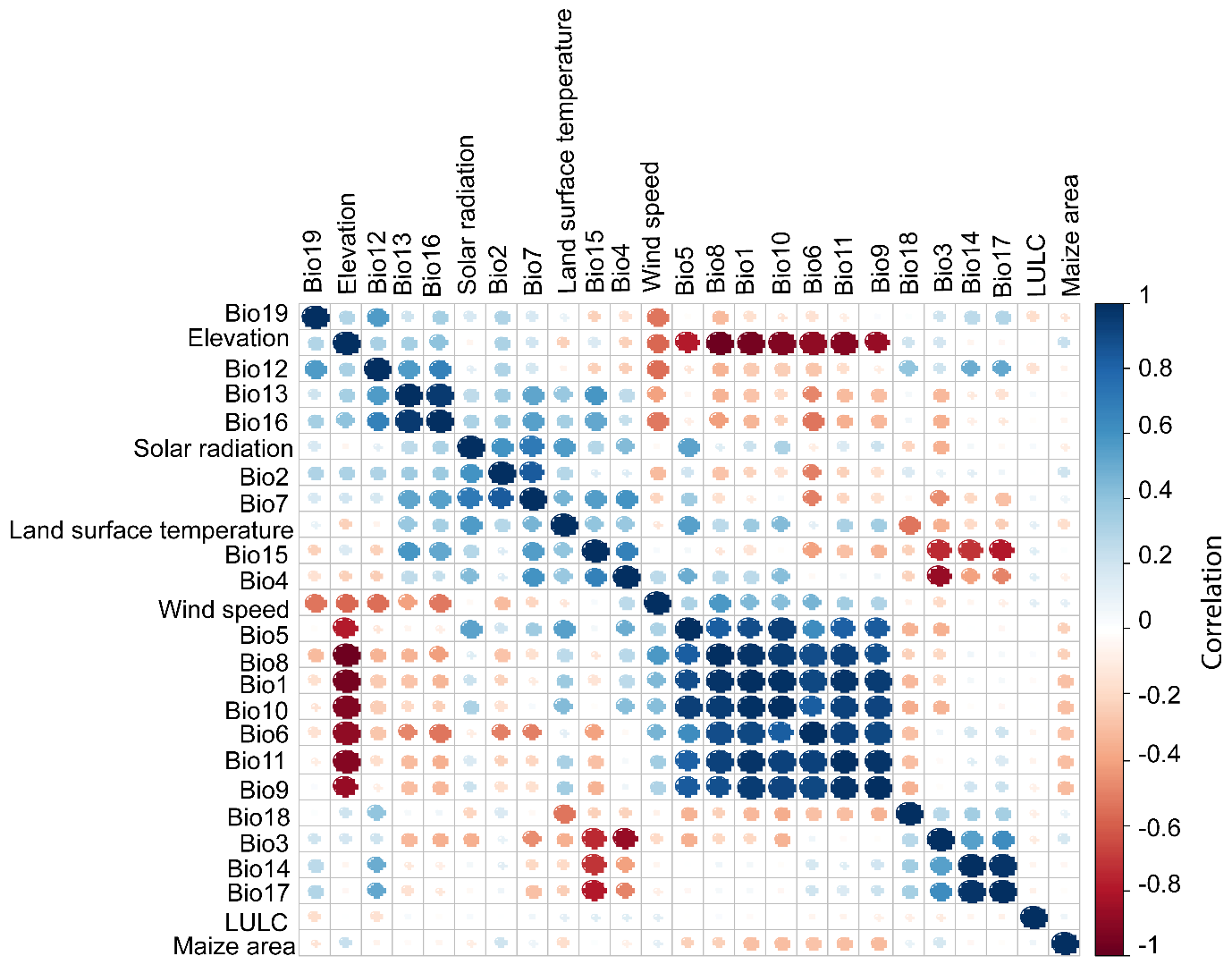


**Supp 2.** Collinearity matrix of the explanatory variables. Darker shades of blue and red spots indicate high collinearity.


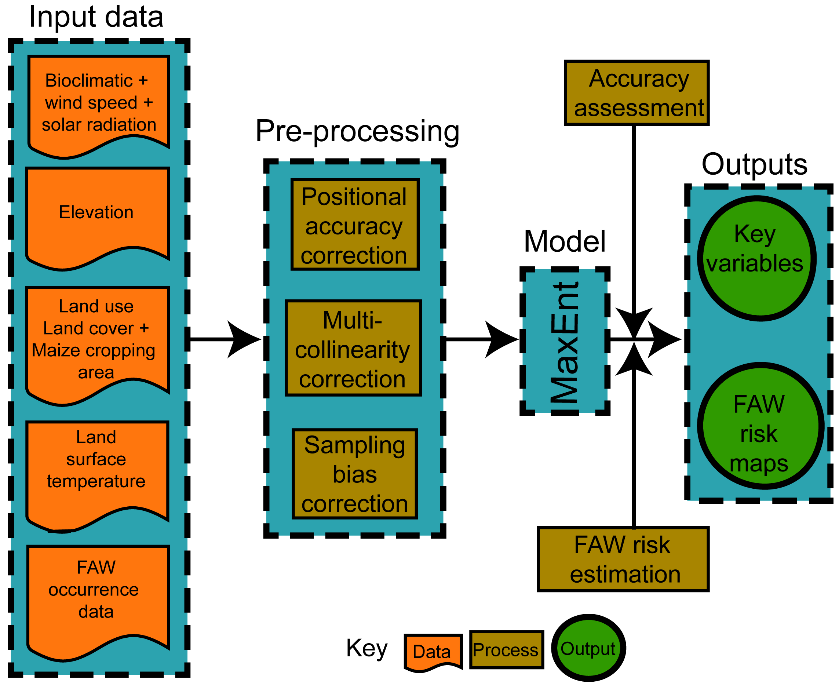


**Supp. 3.** Summarised graphic abstract of the procedure followed in this study to predict fall armyworm (FAW) establishment (risk) in eastern Africa.


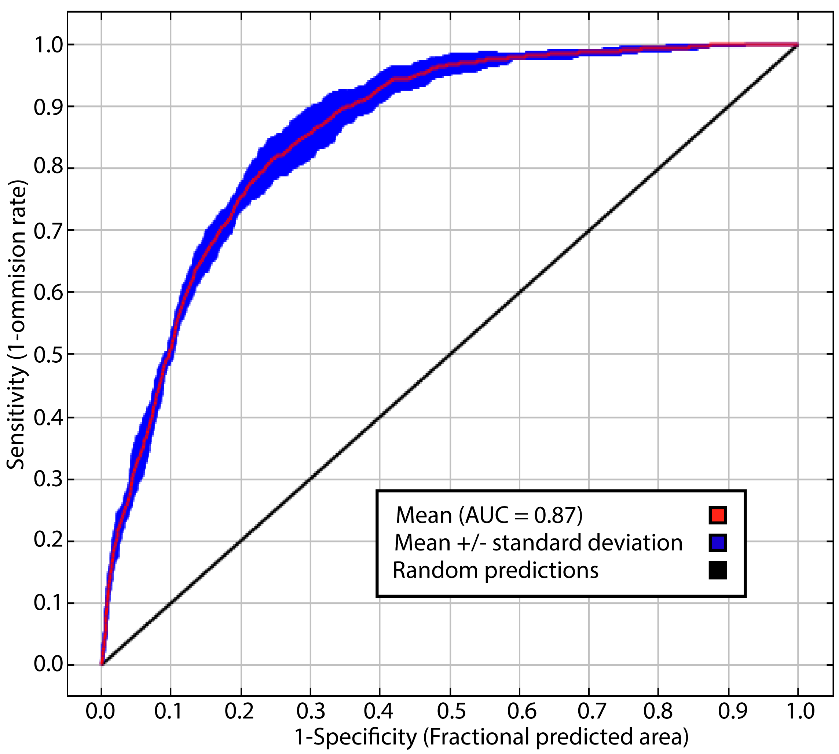


**Supp. 4.** Evaluation of the performance of the replicated MaxEnt models using the area under the curve (AUC). The black straight line demonstrates the predictions performed at random while the red line is the mean plot of the replicated models and the standard deviation of the replicated models is shown by the blue shade


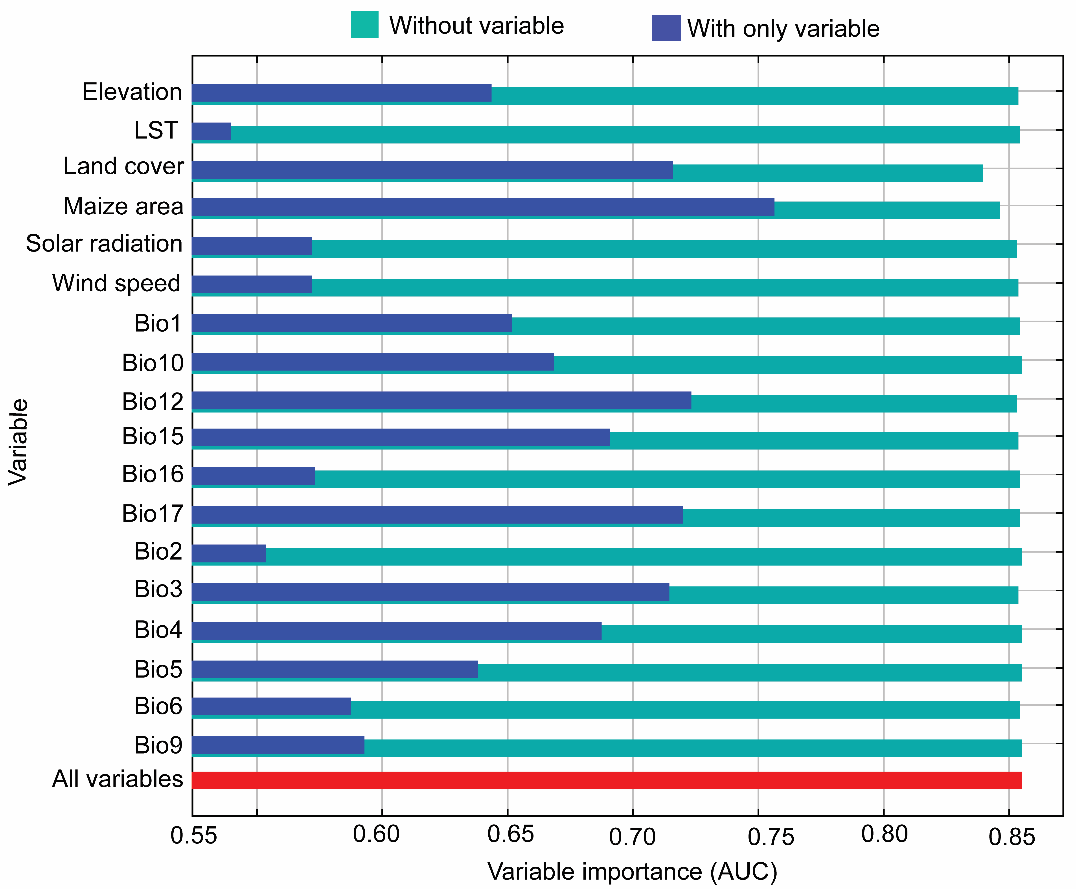


**Supp. 5.** The Jackknife test analysis of the relative relevance of the explanatory variables for simulating the spatial distribution (established habitat) of fall armyworm (FAW).


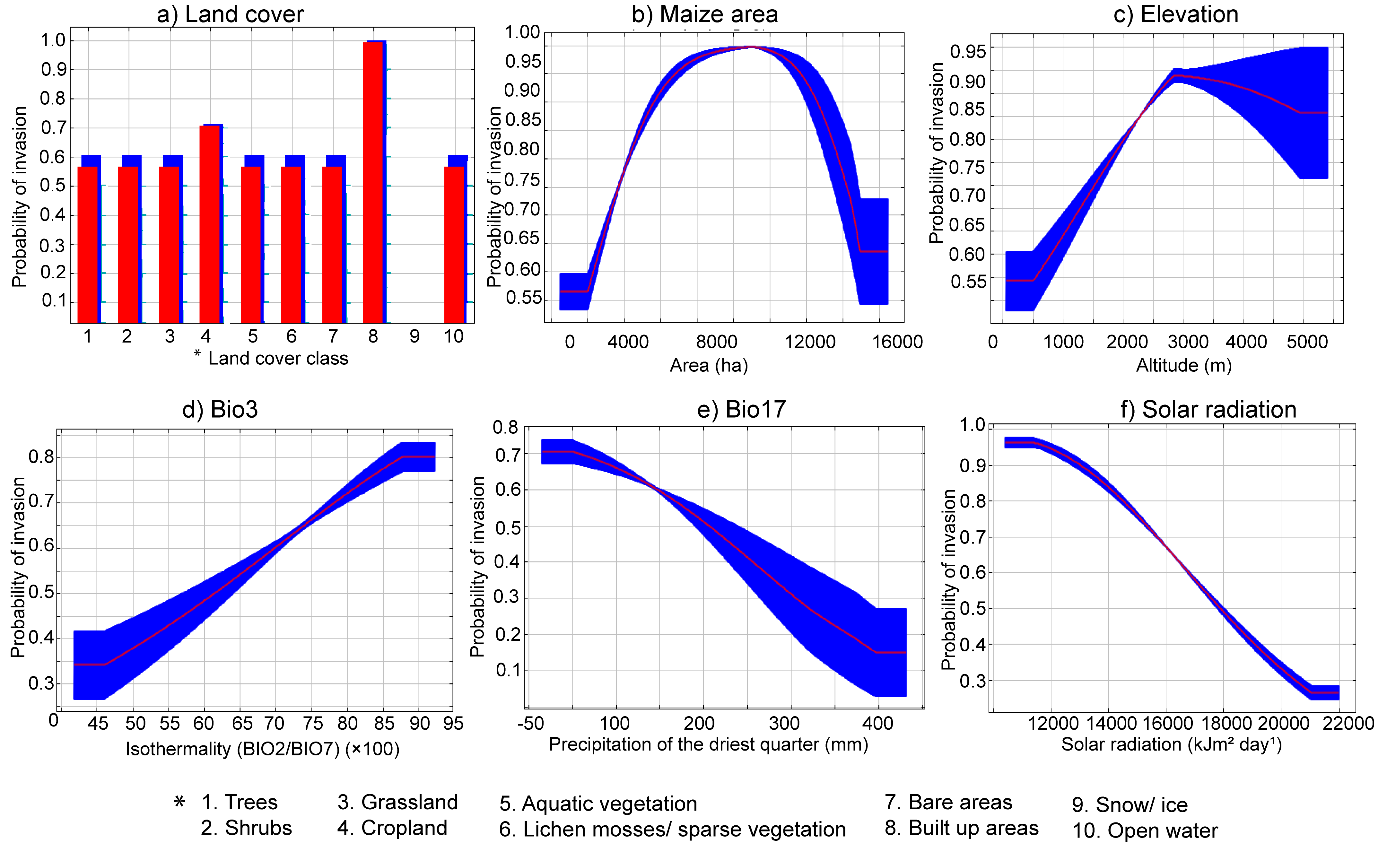


**Supp. 6.** Response curves of the explanatory variables: (a) land use and land cover, (b) maize area, (c) elevation, (d) Bio3 (isothermality), (e) Bio17 (precipitation of driest quarter), and (f) solar radiation on the level of occurrence and establishment of fall armyworm (FAW) in eastern Africa. The red color (curves and bars) in all graphs (a–f) shows the mean of the probability of FAW invasion replicates, while the blue color shows the variance in the replications.
